# A Web-Based Intervention to Improve Health Literacy and Obesogenic Behaviors Among Adolescents: Protocol of a Randomized Pilot Feasibility Study for a Parallel Randomized Controlled Trial

**DOI:** 10.2196/40191

**Published:** 2022-08-16

**Authors:** Sasha A Fleary

**Affiliations:** 1 Department of Community Health and Social Sciences Graduate School of Public Health and Health Policy City University of New York New York, NY United States

**Keywords:** health literacy, adolescent, obesity, prevention, diet, physical activity, web-based intervention, eHealth

## Abstract

**Background:**

Predictive theoretical models suggest that health knowledge works in conjunction with motivation and behavioral skills to influence adolescents’ obesogenic behavior. However, most of the existing adolescent interventions target these variables in isolation. Furthermore, health literacy (HL), a precursor to health knowledge, is necessary for translating health knowledge into behavior and is negatively related to adolescents’ obesity status. However, HL has not been included in obesity interventions targeting adolescents.

**Objective:**

This study aims to pilot the feasibility of a 2-armed web-based obesity prevention intervention in school settings and assess the preliminary effectiveness of adding an HL module to an obesity prevention intervention for adolescents.

**Methods:**

This web-based pilot feasibility study will take place in the Northeastern United States. Participants will be adolescents (aged 13-16 years) attending school, and recruitment will be conducted through flyers to parents and adolescents in participating classes or advisory groups at the school. The intervention includes 2 arms: an experimental arm that will receive an HL module and 3 obesity prevention modules and a comparison arm that will receive a vaping module and 3 obesity prevention modules. A blinded randomized procedure will be used to allocate classrooms and advisory groups to the experimental and comparison arms. The intervention will be fully web-based. Participants will complete measures of their HL and obesogenic behavior–related health knowledge, motivation, and behaviors at 3 time points (baseline, 1 month after the intervention, and 3 months after the intervention) via web-based surveys. The primary outcomes will be the measures of study feasibility (recruitment, retention, completion, and treatment fidelity rates). Secondary outcomes will be preliminary efficacy, as measured by logistic and linear regressions and calculation of effect sizes. Descriptive statistics will be calculated for all measures at each time point.

**Results:**

This study was approved by the City University of New York Institutional Review Board in August 2020. As of June 2022, the web-based intervention design is complete and ready for use. Recruitment, data collection, and intervention implementation are scheduled to begin in September 2022. These results are expected to be published in 2023.

**Conclusions:**

This study’s feasibility findings will inform changes to the intervention content and randomized controlled trial design. The study’s efficacy findings will inform the sample size for the full-scale randomized controlled trial and the preliminary utility of the intervention.

**Trial Registration:**

ClinicalTrials.gov NCT04252677; https://clinicaltrials.gov/ct2/show/NCT04252677

**International Registered Report Identifier (IRRID):**

PRR1-10.2196/40191

## Introduction

### Background

Children and adolescents are increasingly experiencing obesity-related *chronic degenerative diseases*, which were once categorized as adult health problems [1]. For example, in the United States, there was a 30.5% increase in type 2 diabetes diagnoses in adolescents from 2001 to 2009 [2], and more recent data suggest a 7.1% annual increase in diagnoses [3]. A diagnosis of type 2 diabetes in childhood is related to the risk of kidney, nerve, and eye diseases and an increased risk of renal failure and other life-threatening and life-altering complications in young adulthood [4]. Similar to type 2 diabetes, other life-threatening adult chronic health issues such as cancer and heart disease are associated with obesity [5-7] and adolescent health behaviors [8]. Approximately 34% of adolescents in the United States are overweight or obese [9,10]. Furthermore, adolescents’ obesity prevention health behaviors are low: approximately 42% and 41% eat <1 fruit and vegetable daily, respectively, and approximately 77% are physically active for <60 minutes per day [11]. The prevalence rates of fruit and vegetable consumption and sufficient physical activity (PA) are lower among adolescents with low family income [12] and those who identify as racial and ethnic minorities [13]. The prevalence rates of these behaviors must be improved to reduce current and future chronic disease risks in adolescents.

There are several obesity prevention interventions targeting determinants of obesogenic behaviors, including adolescents’ social support and motivation [14-18], behavioral skills [15-18], attitudes [16,18], environment [15,19,20], and health knowledge [15,16]. However, their impacts on adolescents’ obesogenic behaviors have been mixed. Furthermore, most of these interventions did not address all the aforementioned determinants in a single study design. Single determinant–focused interventions likely underestimate the role of individual-context interrelationships, the interrelatedness of the determinants, and the role of adolescent developmental attributes in health decision-making. Importantly, individuals’ decision-making is complex as multiple determinants of health behaviors are either deliberately or unintentionally integrated during the decision-making process. Therefore, although adolescents may prefer or *lean in* on certain types of information and determinants, interventions targeting adolescents’ obesity prevention behaviors should integrate multiple determinants, including knowledge and skills, and incorporate the impact of developmental characteristics and contextual influences on long-term change.

This intervention borrows elements from 3 existing interventions that address multiple determinants in a single design: New Moves [17,21], Go Girls! [16], and the Dutch Obesity Intervention in Teenagers (DOiT) [15]. The school-based New Moves intervention aimed to improve adolescents’ diet- and PA-related knowledge, attitudes, beliefs, skills, and self-efficacy, as well as provide strategies for improving social support [17,21]. The community-based Go Girls! intervention aimed to improve knowledge, self-efficacy, social support, motivation, and behavioral skills for healthy eating and PA [16]. The school-based DOiT intervention aimed to increase adolescents’ knowledge, awareness, behavioral skills, social support, habits, and self-efficacy regarding energy intake and output [15,22]. All 3 studies reported significant postintervention improvements in obesogenic-related behaviors.

These studies included activities that could be adapted for a web-based platform and provided a strong basis for our intervention. However, similar to other adolescent obesity prevention interventions, the New Moves, Go Girls!, and DOiT interventions do not include building adolescents’ general skills for transferring knowledge into behavior (ie, health literacy [HL]). HL “entails people’s knowledge, motivation and competences to access, understand, appraise, and apply health information in order to make judgments and make decisions in everyday life concerning health care, disease prevention and health promotion to maintain or improve quality of life during the life course” [23]. In adults, HL is positively related to engagement in preventive health behaviors, the interpretation of health messages, and medical adherence [24-28]. Although HL is understudied in adolescents, existing research links HL to adolescents’ health behaviors [29] and health decision-making [30,31]. We hypothesize that the inclusion of correlates of health decision-making in health behavior interventions during a critical transitional time to health decision-making independence for adolescents may significantly improve intervention outcomes. To the best of our knowledge, the direct impact of HL on adolescent health behavior change interventions has not yet been examined.

### Objectives

The findings of this pilot study and future full-scale randomized controlled trial (RCT) will inform the inclusion of HL in health behavior interventions for adolescents and the inclusion of HL in the health education curriculum for adolescents. Furthermore, given its digital format, this intervention may easily be disseminated in schools as part of the adolescents’ health curriculum. The main goal of this study is to develop and preliminarily evaluate a web-based obesity prevention intervention for adolescents with and without HL. This study distinguishes between HL and health knowledge; these 2 concepts tend to be inaccurately substituted in some research literature. However, HL is a precursor to health knowledge [32]; that is, HL is the skill needed to access, understand, and use health information for specific behaviors. Despite this, HL is understudied and rarely addressed in health behavior interventions. This study seeks to fill this gap. Aim 1 of this study is to modify and use successful components of existing obesity interventions in an interactive web-based platform with an added-on HL component. Aim 2 of this study is a 2-arm randomized clinical trial of the adapted web-based obesity prevention intervention for adolescents with and without an HL component. The purpose of this pilot RCT is to determine the feasibility and preliminary effectiveness of the intervention in informing the full-scale RCT. Specifically, the primary objectives of this study are to (1) assess the acceptability of the intervention for adolescents; (2) determine the elements of the intervention with the highest adolescent engagement; and (3) determine the suitability and appropriateness of the intervention modality and implementation in school settings by examining the recruitment, retention, completion, and fidelity rates. The secondary objectives of this study are to measure the preliminary efficacy of the intervention to improve obesogenic behaviors and HL and collect data to calculate effect sizes and power analyses to inform the sample size needed for the full-scale RCT to determine whether adding an HL component to a web-based obesity prevention intervention improves adolescents’ obesity prevention behaviors more than an obesity prevention intervention alone.

## Methods

### Trial Design

**Figure 1:**
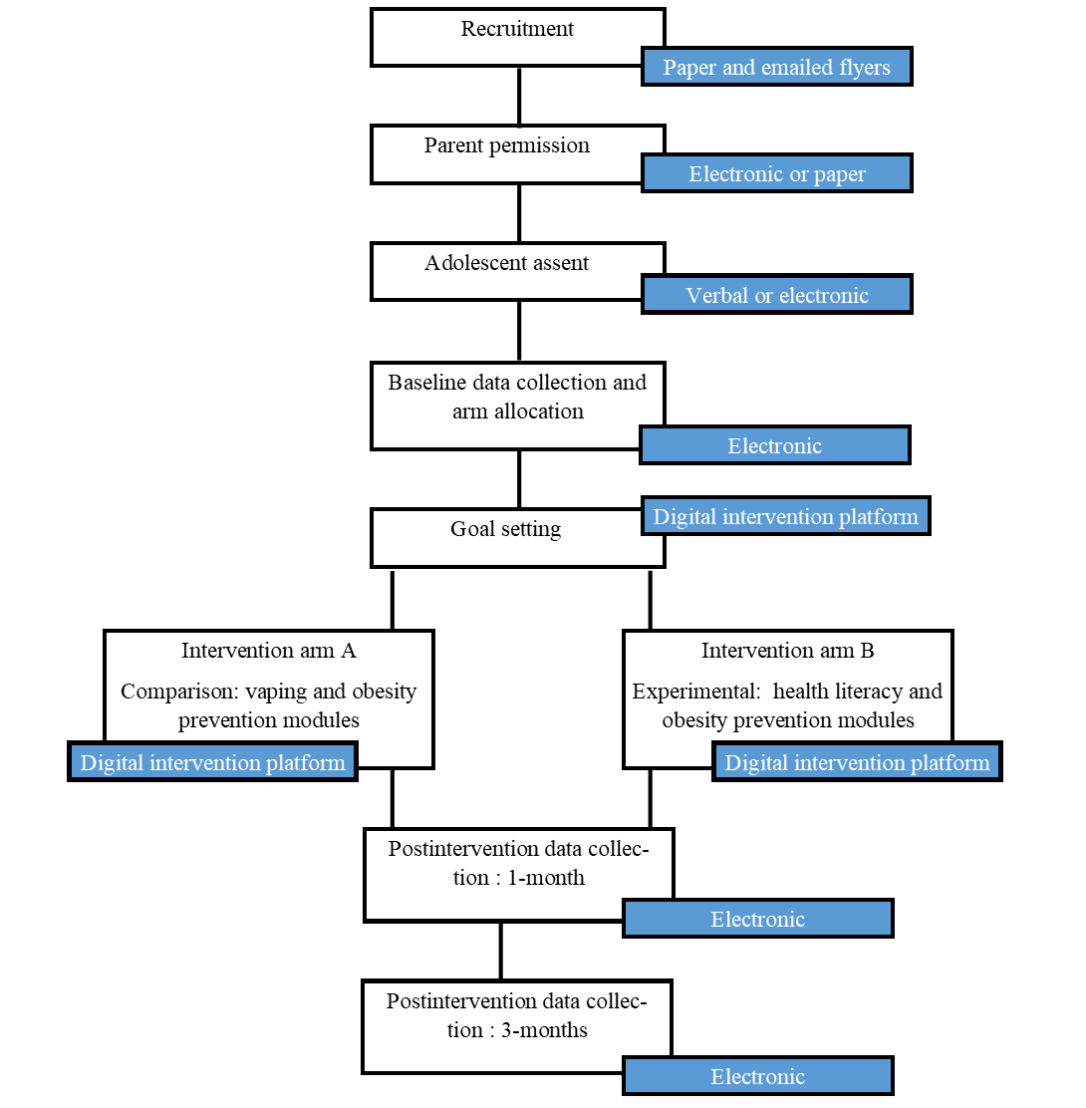
Study procedures flowchart.

The trial design elements are reported to be consistent with the CONSORT (Consolidated Standards for Reporting Trials) guidance for pilot and feasibility studies (Multimedia Appendix 1) [33] and the CONSORT-EHEALTH (Consolidated Standards for Reporting Trials of Electronic and Mobile Health Applications and Online Telehealth; Multimedia Appendix 2) [34]. This study is a parallel randomized controlled pilot trial and feasibility study with 1:1 classroom allocation to 2 study arms (experimental and comparison groups). Figure 1 shows the outline of the study flow.

### Participants

A total of 76 adolescents aged between 13 and 16 years attending high school in Boston, Massachusetts, United States, will be recruited for the study. The other inclusion criterion is parental permission to participate. Adolescents who are already participating in interventions related to healthy eating, PA, and obesity prevention or treatment or who have medical conditions that prevent them from engaging in PA or require adherence to extremely restricted diets (eg, ketogenic diet) will be excluded from the study. The proposed sample distribution for the study is at least 30 male and 30 female adolescents so that comparisons can be made across sexes, given the difference in obesogenic behaviors based on sex. In addition, 80% of the sample is expected to come from racial and ethnic minority groups, given that the study will be conducted in a multiethnic geographic location and school setting.

### Recruitment and Consent

Responsive survey design methodology [35] will be used to ensure that the proposed sample is recruited. Specifically, we will monitor participant enrollment and establish timelines for changing or adding recruitment strategies to ensure that the proposed sample is recruited. In responsive survey design methodology, the least costly recruitment strategy is used first, and more expensive and time-consuming strategies are used to augment the inexpensive strategies to enroll hard-to-recruit participants. For the first phase of recruitment, school administrators will give study flyers to students and email them to parents of students in participating classes. The emailed flyers will include a Qualtrics link (Qualtrics) for parents to access the web-based parent permission form and a brief demographic survey. Parents will select *yes* or *no* to indicate whether they agree for their adolescent to participate in the study. The class or advisory group with the highest rate of returned parent forms (regardless of whether permission was granted or denied) will receive a class prize (eg, donation to the class field trip fund) at the end of the first 2 weeks of the recruitment period. The characteristics of the enrolled participants will be monitored to determine progress in recruiting the desired sample with regard to sex and racial and ethnic minority status. At the 2-week time point during phase 1 recruitment, the second phase of recruitment will be initiated in an attempt to achieve the desired sample. In phase 2, adolescents whose parents did not complete the permission form on the web will be given a study flyer with a QR code for parents to access the Qualtrics link. A paper permission form, demographic survey, and an empty envelope will also be attached to the flyer. Parents who complete the paper permission form will be asked to put it in the envelope, seal the envelope, and have their adolescents return the envelope to their teacher. At each phase of recruitment, teachers will also make announcements about the study in class, encouraging students to have their parents complete the permission forms. Recruitment will end 2 weeks after the initiation of phase 2 recruitment. The class with the highest increase in returned consent forms at the end of the second phase of recruitment will be incentivized with a class prize (eg, donations to their class trip and dance). The phase 1 recruitment strategy will be ongoing throughout the 4 weeks of recruitment as parents will be sent weekly reminder emails to complete the permission form and demographic survey. These procedures are consistent with several national studies (eg, the National Survey of Family Growth [36,37] and the National Survey of College Graduates [38]). Active monitoring of participant enrollment will occur throughout the 4 weeks of study recruitment and enrollment. To ensure that participation is voluntary and not coerced, incentives will be attached to the return of consent forms rather than study enrollment. All recruitment and consent materials will be available in English, Spanish, and Haitian Creole to accommodate Haitian Creole–speaking and Spanish-speaking parents with limited English language proficiency. Adolescent assent will be obtained on the web before data collection on the first page of the web-based survey. Adolescents who select *no* to assent will be exited out of the survey, and those who select *yes* will be able to see the survey. It should be noted that parental permission and adolescent assent are specific to the data collection related to the study. Adolescents who do not assent or obtain parental permission will still be required by their teachers to participate in the intervention as part of their class curriculum but will not complete surveys.

### Intervention

Both study arms will receive 3 obesity prevention modules. However, the first study arm (ie, the comparison group) will also receive 1 module on vaping, whereas the second study arm (ie, the experimental group) will receive a module on HL. Obesity prevention modules will be administered after the vaping and HL modules. The intervention modules will be delivered using a web-based platform. The platform allows for a mix of didactic strategies, including informational videos, interactive practice activities and games, and self-assessments. Responses to assessments, activities, and games will be used to provide tailored feedback and reports. The intervention will be fully web based. Table 1 presents the objectives of the intervention modules, lesson objectives, and sample activities. It should be noted that all content was developed by the author and her team, and the content is hosted using the Dynamic e-Learning Platform, a web-based platform developed by the 3C Institute [39], which uses a mix of didactic instructions with self-assessments, demonstration videos, and interactive practice activities to apply learned skills.

**Table 1:** Intervention module and lesson objectives and sample activities^a^.

Modules or lessons		Objectives	Activities
Introduction		N/A^b^	N/A
Goal planning		To guide adolescents as they make one eating and one physical activity goal that is specific, measurable, attainable, relevant, and time based	Initial: assessment of adolescents’ current dietary intake and physical activities; tailored suggestions for goals are provided based on adolescents’ responses; adolescents then pick the goal they want to work on and are guided on making a plan for their goal through a series of how, when, where, and who questions with suggestions for responses Other: check-in about progress on goals at the end of each module with tailored tips for getting back on track or increasing intensity of their goals based on their responses; tips correspond with the module (eg, tips after the motivation module focus on personal motivation and social support)
**Getting the Skills Down module (HL^c^;** **experimental group only)**		To improve adolescents’ functional, interactive, critical, and media HL	N/A
	Lesson 1: Reading Labels (functional HL)	To demonstrate how to read and use nutrition and medicine labels	Didactic videos explaining the information on the labels with a focus on reading comprehension and numerical calculations, followed by practice exercises, including labels and scenarios in which adolescents would use labels
	Lesson 2: Talking to Other People About Health (interactive HL)	To provide and reinforce skills for gathering health information from others and discussing health concerns with others, including friends, family, and health care providers	Demonstration videos providing instructions and examples on how to talk to different people about health, followed by activities and scenarios to practice preparing for and navigating health conversations with parents, friends, providers, and other trusted adults
	Lesson 3: Choosing Good Sources for Health Information (media HL)	To equip adolescents with skills to evaluate sources of information for reliability, accuracy, and intent and teach adolescents how to use multiple sources of information in health decision-making	Didactic demonstration videos explaining how to identify and evaluate good sources of health information, followed by activities and scenarios where adolescents identify and evaluate good sources of information
	Lesson 4: Using Health Information to Help Yourself and Others (critical HL)	To provide and reinforce skills for applying health information to improve personal and community health, educate adolescents about the role of social determinants of health in health decision-making, and provide suggestions on how to advocate for others	Demonstration videos with illustrated scenarios showing different outcomes to different health decisions and didactic videos on how social determinants of health affect access to health resources and health decision-making and on advocacy; videos followed by activities evaluating the social environment for health, self-reflection to identify skills and passions, and how adolescents may want to use it to change their community
**Getting the Truth module (vaping;** **comparison group only)**		To improve adolescents’ knowledge of the effects of vaping and provide strategies for preventing and seeking help when vaping is implicated	N/A
	Lesson 1: Vaping Devices and Vaping	To explain what vaping is, how vapes work, and the effect of vaping on the body	Demonstration videos on how vapes work and how it affects the body and people around the vaping person, followed by activities to reinforce knowledge about how vapes work and the consequences of vaping; quizzes to test knowledge about vaping administered before and after lessons
	Lesson 2: Beliefs and Attitudes about Nicotine Products	To challenge common beliefs and attitudes about nicotine products and vaping specifically	Demonstration videos showing the consequences of vaping and debunking common myths about vaping, followed by reflection questions about adolescents’ beliefs about vaping and interactive activities where adolescents distinguish beliefs that may be myths vs facts
	Lesson 3: Refusing and Avoiding Vaping	To suggest strategies for refusing offers to vape and avoiding situations where friends vape	Didactic videos demonstrating tips for refusing to vape and avoiding vaping, followed by activities and scenarios to practice conversations with friends to handle situations where vapes are present
	Lesson 4: Recognizing Addiction and Getting Help	To provide tips on how to recognize when oneself or a peer is addicted to vaping and how to get help	Didactic videos describing why and when some adolescents would want to receive help and where to receive help, followed by activities and scenarios where adolescents make decisions about receiving help
**Getting the Facts Straight module**		To improve adolescents’ health knowledge about obesity prevention, healthy eating, physical activity, and sedentary behavior	N/A
	Lesson 1: The Facts about Healthy Eating	To improve and reinforce adolescents’ knowledge about what is required in a healthy diet and how to distinguish between healthy and unhealthy foods	Didactic videos explaining healthy and unhealthy diets, followed by activities and scenarios around identifying and making healthy and unhealthy diet choices
	Lesson 2: The Facts about Physical Activity	To inform adolescents about the recommendations for different types of physical activity and what is considered physical activity	Didactic videos about the types of physical activity and the FITT^d^ principle recommendations, followed by scenarios to practice different aspects of the FITT principles and guided exercises to help adolescents make a FITT plan for themselves
	Lesson 3: Eating, Exercise, and Mood	To help adolescents recognize the connections between their eating, physical activity, and mood and provide proactive strategies to ensure that mood is not negatively affected by poor eating and activity choices	Didactic videos describing the relationship between eating, physical activity, and stress, followed by activities on the effect of skipping meals and physical activity on mood and scenarios involving decision-making around scheduling meals and activities to avoid negative effects on mood
**Getting the Mind Ready module**		To improve adolescents’ personal and social motivations for engaging in obesity prevention behaviors	N/A
	Lesson 1: What Motivates You?	To encourage adolescents to think about what may motivate them to engage in healthy eating and physical activity and provide opportunities for self-reflection on what motivates adolescents	Didactic videos describing motivation and providing examples of what motivates different adolescents and tips for getting motivated, followed by self-reflection activities and scenarios with adolescents needing the motivation to engage in healthy eating or physical activity
	Lesson 2: Social Support	To describe how people in adolescents’ lives can support their health goals and provide strategies for identifying and using sources of social support	Didactic videos describing the types of social support and how adolescents may use these support to achieve their health goals, followed by reflection questions on the types of support adolescents already have and want and scenarios where adolescents practice navigating social support
	Lesson 3: You Can Do This!	To provide adolescents with practical strategies for staying motivated	Demonstration videos on setting small goals, finding goal partners, and self-efficacy, followed by scenarios to help characters set SMART^e^ goals and identify goal partners and reflection questions to help participants identify their goal partner’s needs and options
**Get Going module**		To address socioeconomic (and other) barriers to preventive health by reinforcing doable behavioral skills within the socioeconomic constraints experienced by adolescents	N/A
	Lesson 1: Setting Yourself Up to Make Healthy Choices	To provide adolescents with skills for making healthy eating and physical activity choices from the options available to them	Demonstration videos demonstrating meal planning, grocery shopping, and identifying and using physical activity when one has limited resources, followed by activities to identify physical activity opportunities in different situations and settings and scenarios for healthy eating where adolescents have limited resources
	Lesson 2: Using the 3 Rs to Make Healthy Choices	To explain how adolescents may use the 3 Rs (replace, reduce, and remove) when making decisions about healthy eating and physical activity	Demonstration videos showing the 3 Rs in practice and providing tips for using the 3 Rs, followed by activities and scenarios to practice using the 3 Rs
	Lesson 3: Practice Making Healthy Choices	To practice knowledge and skills learned throughout the 3 obesity modules in the intervention	A game where the participant chooses an avatar (adolescent) and helps the avatar make healthy decisions from the time they wake up to the time they go to sleep; tailored feedback is provided after each decision

^a^Tailored feedback was provided based on adolescents’ responses to the activities and questions throughout the intervention.

^b^N/A: not applicable.

^c^HL: health literacy.

^d^FITT: frequency, intensity, type, and time.

^e^SMART: Specific, Measurable, Achievable, Relevant, and Time-bound.

**Figure 2:**
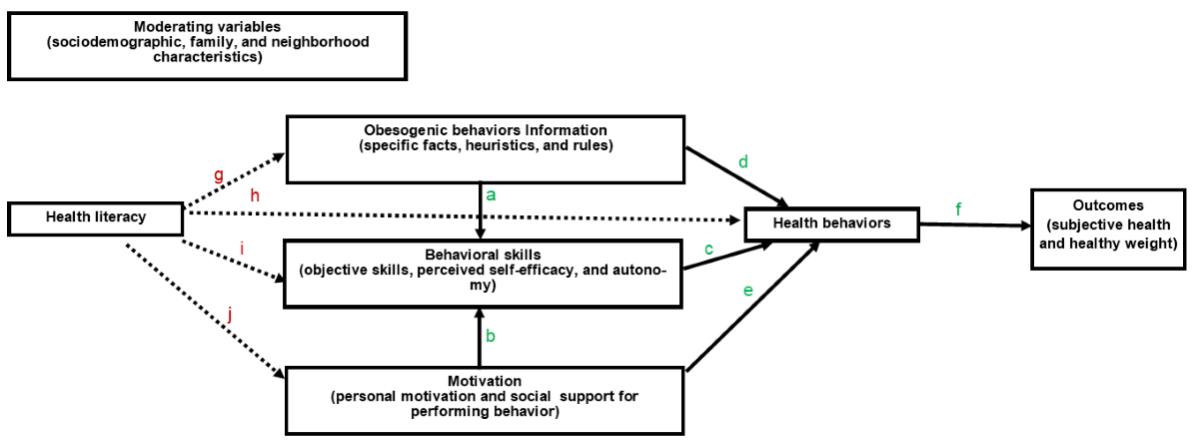
Information-Motivation-Behavioral Skills theoretical model.

This intervention is informed by the relational developmental systems framework, the Information-Motivation-Behavioral Skills (IMB) model, adolescents’ inputs, and prior studies. The relational developmental systems framework, which is a developmental science framework, centers on the mutually influential relationships between individuals and contexts, as well as the plasticity of the relationships as the individual or context changes [40]. Research that applies a relational developmental systems framework seeks to answer *what important characteristics of individuals (eg, motivation, autonomy, health identity, health knowledge, and HL), among individuals of what status (eg, adolescents and low income), and in relation to what elements of the context (eg, family, peers, and neighborhood) are likely to be associated with what aspects of adaptive functioning (eg, healthy eating and PA).* As such, this intervention is designed to account for and intervene in the mutually influential relationship of adolescents’ individual and developmental characteristics, social determinants of health, health knowledge, and HL on health behaviors. The relational developmental systems framework is complementary to the more predictive IMB model [41]. The IMB model includes 3 critical determinants of the performance of health behaviors: health-related information (facts and beliefs), motivation (personal attitudes and social support), and behavioral skills (objective skills and confidence in performing the behavior). Information and motivation support behavioral skills that influence behavior (Figure 2, arrows *a*-*c*). However, information and motivation may directly affect behavior when only basic skills are required (Figure 2, arrows *d*-*e*). Health behaviors ultimately predict health outcomes (Figure 2, arrow *f*).

The relational developmental systems framework was used to determine the variables that were explored in the IMB model. Given that the health-related information and behavioral skills constructs in the IMB model are specific to health behavior and that HL is a precursor to health knowledge [32] and likely independently influences motivation, behavioral skills, and behavior, HL was included as a separate construct in the model (dashed lines in Figure 2). We expect HL to directly predict information, motivation, behavioral skills, and health behaviors (Figure 2, arrows *g*-*j*) and indirectly predict health behaviors through information (g→d and g→a→c), motivation (j→e and j→b→c), and behavioral skills (i→c). Therefore, improvements are expected in the experimental conditions for all variables in the model compared with the comparison group.

Regarding specific content, initial focus groups with adolescents informed the initial content of the HL modules, whereas prior obesity prevention intervention studies informed the content for the obesity prevention modules. In a preintervention development study by the author, adolescents participated in multiple focus groups where they described how they viewed HL, how they knew they were using HL, and what type of HL skills they felt they lacked [30,31]. Analyses of adolescents’ responses resulted in the development of the IMB model for the use of HL in health decision-making in adolescents [30]. The conceptual underpinnings of the IMB model for the use of HL in health decision-making in adolescents and specific focus group findings were used to determine which key features of HL should be included in the intervention and how to present the content in a way that matches the context in which adolescents view themselves as engaging in HL. The obesity prevention modules were informed by the successful components of New Moves [17,21], Go Girls! [16], and DOiT [15].

Regarding acceptability and usability, adolescents participated in cognitive interviews while completing the HL and behavior skills modules. This was done before building out the other modules of the intervention. The feedback provided on the HL and behavior skills modules was used to revise the modules and was applied to the development of the remaining intervention modules.

For this feasibility study, all adolescents in the participating classes or advisory group will access the intervention on school computers during their homeroom or advisory periods. The intervention will be provided in lieu of class activities and will be solely web based. Specifically, teachers will only be involved in instructing adolescents to log into the web-based platform, and all other activities of the intervention will occur via the web-based platform. Adolescents will complete 1 lesson per homeroom or advisory period with 1 to 2 lessons per week, depending on their class schedule. The intervention website includes a *contact us* button so that students can report any concerns or comments. This will be closely monitored by research staff, who will work to resolve issues as quickly as possible.

### Outcomes

#### Primary Feasibility Outcome Measures

According to Bowen et al [38], the assessment of study feasibility involves answering 3 main questions across 8 key areas of focus. Although Bowen et al [38] suggested asking whether it can work, does work, and will work, this feasibility study mostly focused on the first 2 questions (whether it can work and does work). The third question (whether it will work) is better aligned with the activities of a full-scale RCT. Textbox 1 provides a further explanation of the 8 areas of focus, as they relate to this study.

**Feasibility as applied to this study.**

**Acceptability: satisfaction, suitability, and attractiveness of the intervention**

- Can it work?: In the prepilot activities, qualitative feedback on session components was solicited and used to modify the intervention for suitability and attractiveness.
- Does it work?: The pilot intervention will assess recruitment, completion, retention, and treatment fidelity rates as measures of satisfaction, suitability, and attractiveness of intervention.

**Demand: likeliness of using the intervention**

- Can it work?: In the prepilot activities, adolescents’ risk and protective factors for obesogenic behaviors were considered in intervention development to ensure that the skills reinforced in the intervention would be generalizable across settings.
- Does it work?: The longitudinal study design will provide preliminary data on the retention of skills learned in the intervention. Web analytic data collected during the intervention (eg, time spent on modules and resources accessed through intervention) will determine the likeliness of using the intervention.

**Implementation: successful delivery**

- Can it work?: In the prepilot activities, participants were observed engaging with intervention content, and their comments were used to make content and stylistic changes to improve the utility and likelihood of prolonged engagement with the content.
- Does it work?: A pre-post follow-up design allows for the determination of the 3-month success rate of the intervention in changing obesity-related knowledge, motivation, and behavior. Participants’ progress on goals will also be a marker of success.

**Practicality: deliver intervention within confines of current resources**

- Can it work?: Participants’ usability observations and intervention evaluations will be used to assess feasibility, with participants serving as key informants.
- Does it work?: Postmodule evaluations will provide data to inform the next steps to improve the reach and effectiveness of the intervention (eg, need for tangible support such as fitness videos and fresh food boxes). Cost analyses will be conducted.

**Expansion: expand to provide new service**

- Can it work and does it work?: For future project goals and next steps, a randomized controlled trial with a representative sample powered to detect significant changes should be implemented. Future projects should also expand to include language and other cultural adaptations for specific groups (eg, Mexican, Haitian, and Dominican).

**Limited efficacy: potential for success in a controlled environment**

- Does it work?: The pilot design allows for assessing the potential for success in a controlled environment and calculation of effect size to develop larger efficacy trials.

**Adaptation: implementation in new population**

- Can it work and does it work?: For future project goals and next steps, the effectiveness of intervention in other settings (eg, libraries) need to be assessed, and feasibility and effectiveness in different populations after cultural and language modifications need to be evaluated.

**Integration: successful integration into existing settings**

- Can it work and does it work?: For future project goals and implications, the intervention should be integrated into schools and community organizations, and the sustainability in its current form must be monitored.

#### Secondary and Preliminary Efficacy Outcome Measures

Participants’ demographics (eg, age, sex, gender, family income, race, ethnicity, and grades) and other characteristics (eg, home and neighborhood environment characteristics) will be collected at baseline. Secondary measures will be collected at each time point and will serve as measures of the preliminary efficacy of the intervention. Table 2 outlines the secondary measures used in this study.

**Table 2:** Secondary preliminary efficacy measures^a^.

Measure	Topics assessed	Psychometric properties
Dietary screener questionnaire	Healthy and unhealthy food consumption	Good convergence with 24-hour dietary recalls [42]
National Youth Physical Activity and Nutrition Study	Healthy and unhealthy food consumption	Validity and reliability established [43,44]
Youth activity profile	Physical and sedentary activity	Cross-validated with objective measures of physical activity and resulted in similar group estimates acquired from objective measures [45]
Godin and Shephard Leisure-Time Physical Activity Questionnaire	Metabolic equivalents for leisure time physical activity	Validity and reliability established [46]
Newest vital signs	Functional health literacy	Cronbach α=.76; criterion validity *r*=0.59 [47]
Assessments of adolescent health literacy	Functional, interactive, and critical health literacy	Wright sample-independent reliability ≥0.80; good convergent and criterion validity [48]
Adolescent media health literacy scales	Media health literacy	Wright sample-independent reliability ≥0.80; good convergent and criterion validity [49]
Electronic Health Literacy	Digital health literacy	Cronbach α=.88 [50]
Canadian Assessment of Physical Literacy (modified for the study)	Physical activity knowledge	Validity and reliability established across multiple groups [51]
General Nutrition Questionnaire for Adults (modified for the study)	Healthy eating knowledge	Cronbach α≥.70; good construct validity [52]
Regulation of Eating Behaviors Scale (modified for the study)	Healthy eating attitudes	Cronbach α≥.79; good construct validity [53]
Psychosocial constructs for adolescent fruit and vegetables and dietary fat intake	Social motivation for healthy and unhealthy eating; diet-related self-efficacy	Cronbach α≥.61 [54]
Psychosocial constructs for adolescent physical activity and sedentary behavior	Social motivation for physical and sedentary activity; physical activity self-efficacy	Cronbach α≥.67 [55]

^a^All measures will be administered at baseline, posttest, and 3-month follow-up time points.

### Data Collection

Data collection will be completed on the web using the Qualtrics survey platform. After a team member verbally explains the assent form, adolescents will be provided with a link where the first page will include the written assent form. After providing assent, adolescents will be routed to the survey. For the posttest and follow-up measures, adolescents will complete surveys 1 month and 3 months after the completion of the intervention. Similar to the baseline, data collection will be completed on the web using the Qualtrics survey platform.

Adolescents will be randomly selected to complete qualitative interviews at the end of the intervention to gather feedback on usability, acceptability, and suggestions for changes. All data collection will be performed while the adolescents are in school. Data files and other electronic study records will be stored in password-protected folders on a restricted-use server. Identifiable information will be kept separate from survey data; identifiable information will be deleted after the 3-month follow-up data collection is complete, and the data will be linked to the 2 other time points. Paper study materials will be scanned, and the scanned files will be stored in the same way as the electronic data. The paper study materials will be shredded after they are scanned.

### Sample Size

The sample size was based on power calculations computed using the preliminary data. Preliminary data suggested that a sample of 60 participants (30 per group) would be sufficient. We plan to enroll and randomize 76 participants to allow a 20% dropout rate at the 3-month follow-up. The sample size is sufficient to calculate feasibility metrics and preliminary efficacy and inform future power calculations for a more complex full-scale RCT.

### Randomization and Blinding

#### Sequence Generation

Classrooms and advisory groups will be randomly assigned to the experimental and comparison groups, with 2 classrooms and advisory groups per condition. Simple randomization will be performed using a random number computer program. Specifically, a blinded research team member who is not involved in providing assent and has no knowledge of the students in the classes and advisory groups will assign each classroom and advisory group a number from 1 to 4, and then, a computerized random number generator will be used to assign the classroom and advisory group to the conditions.

#### Allocation Concealment Mechanism

Randomization and intervention condition allocation will occur after the assent and baseline measure data collection. This information will be communicated to the teachers so that they are aware of the content that the adolescents will receive. It is not possible to conceal allocation to adolescents as it will be obvious whether they received the HL or vaping modules.

#### Blinding

Participants will not be explicitly made aware of whether they are in the experimental or comparison group. However, it is not possible to *blind* them to their condition as the experimental condition is obvious.

### Statistical Methods

#### Data Analysis

Regarding the metrics mentioned in Textbox 1, the recruitment rate will be assessed by calculating the percentage of adolescents enrolled in the study from the pool of adolescents approached for participation. The completion rate will be assessed by calculating the percentage of adolescents who complete the intervention from those who enroll. Retention rates will be assessed by calculating the percentage of participants who complete all elements of the study (intervention and data collection) compared with the number of participants who enroll. Treatment fidelity will be assessed using web analytics data gathered during the intervention. The median time spent on each intervention task will be calculated, and participants who spend <3 median absolute deviations below the median will be assumed to have not engaged with the content enough to be fully exposed to the treatment. Treatment fidelity percentages will be calculated based on users meeting a priori thresholds. On the basis of intervention research in adolescents [56-58], the conservative acceptable targets are as follows: recruitment=50%, completion=70%, retention=50%, and treatment fidelity=80%. Descriptive and content analyses will be conducted for quantitative and qualitative evaluation data, respectively.

Descriptive analyses will be calculated for all variables in Table 2. The 2 study groups will be compared in terms of baseline variables to assess whether a balance was achieved through randomization. Changes in continuous outcome variables from the pretest time point to 3 months will be compared between groups using linear regression, adjusted for sex and any potential confounders that are found to be unbalanced between groups at baseline. Binary outcome variables (outcome improved vs not improved) will be compared between groups using logistic regression, adjusted for sex and any potential confounders that are found to be unbalanced between groups at baseline. For each outcome, we will also analyze the time points together in the same model using linear and logistic mixed-effects models with random subject-specific intercepts and slopes. This will permit the analysis of all participants, including those who drop out. Mixed models can accommodate missingness at random.

Group differences in the change in metabolic equivalents and number of fruits and vegetables, as well as the SDs of the changes in each group, will be used to calculate the sample size for the full-scale RCT.

#### Power

This study is powered based on fruit and vegetable consumption and PA outcomes. Assuming 30 participants per group, an independent-sample *2*-sided *t* test, within-subject correlation of 0.5, and α=.025, there will be 80% power to detect a difference between groups on the change in the metabolic equivalents (PA) and fruit and vegetable consumption from the pretest time point to the 3-month follow-up of whether the true difference is 0.8 (PA) or 1.2 (fruits and vegetables) SDs or larger (ie, effect size 0.8). These calculations are based on preliminary data [59]. The data used in this study will be used to perform the power calculations.

### Ethics Approval and Consent to Participate

All study procedures were approved by the City University of New York Institutional Review Board (2020-0575-PHHP) and will be conducted in accordance with the approved application. Data will only be used from participants with signed parent permission forms and who will assent to participate.

## Results

The study has received institutional review board approval. Recruitment for the trial has not yet begun. Data collection is expected to begin in September 2022. The results are expected to be submitted for publication in an updated manuscript in June 2023. Feasibility and primary efficacy results will be reported according to the CONSORT guidelines [33] after the 3-month follow-up data are collected and analyzed.

## Discussion

### Principal Findings

Interventions to improve adolescents’ health behaviors have yielded mixed results, and HL may be a key missing element in improving the efficacy and effectiveness of existing interventions. However, to the best of our knowledge, this is the first intervention study to test the utility of adding HL to health behavior interventions for adolescents. The findings of this study will inform the inclusion of HL as an intervention component, as well as the extent to which the principles of HL and adolescents’ HL levels are considered in interventions designed for adolescents. Furthermore, the use of a digital platform (rather than in person) to implement the intervention is novel, and this study will provide evidence and preliminary data for using digital platforms for HL interventions. This is critical as interventions using digital platforms extend the reach and use of the intervention modules, as geography, personnel, and time constraints minimally affect implementation.

As an intervention similar to this one has not been conducted before, the range of feasibility constructs assessed in this pilot RCT will determine the optimal procedures for the full-scale RCT, as well as the dissemination of the intervention beyond the full-scale RCT. The information gathered from the feasibility constructs will also inform how other similar research questions are addressed in similar settings. For example, a digital intervention on adolescent substance use prevention and HL may use a similar methodology. Obtaining objective fidelity measures provides useful data on what might work in the intervention format beyond the subjective evaluation tools.

### Limitations

A strength of this pilot and feasibility study is the inclusion of outcome measures to facilitate power calculations for a full-scale RCT. However, the small sample size means that preliminary efficacy is mostly useful for calculating RCT sample needs rather than making strong inferences about the utility of the intervention. Another limitation of this study is the use of schools. Schools are an isolated portion of the adolescent population as they exclude adolescents who may be homeschooled or out of school for other reasons. These excluded adolescents may have different HL, health behavior change needs, and access to resources; therefore, the efficacy and effectiveness of the intervention for these adolescents cannot be determined from this study. Furthermore, blinding could not be implemented. This may affect the results as adolescents in different conditions may discuss and compare what they are doing in their conditions, and this can lead to information seeking and knowledge acquisition, which conflates the findings.

### Conclusions

This study is the first step toward addressing the HL needs of adolescents within the context of health behavior interventions. The findings of this study could inform the design and content of future health behavior interventions designed for adolescents.

## Appendix

Multimedia Appendix 1 CONSORT (Consolidated Standards for Reporting Trials) 2010 extension for pilot and feasibility trials checklist.

Multimedia Appendix 2 CONSORT-eHEALTH (Consolidated Standards for Reporting Trials of Electronic and Mobile Health Applications and Online Telehealth) checklist (V 1.6.1).

Multimedia Appendix 3 Peer review by the Center for Scientific Review Special Emphasis Panel - Small Grants for New Investigators to Promote Diversity in Health-Related Research (National Institutes of Health, USA).

## Abbreviations

CONSORT: Consolidated Standards for Reporting Trials
CONSORT-EHEALTH: Consolidated Standards for Reporting Trials of Electronic and Mobile Health Applications and Online Telehealth
DOiT: Dutch Obesity Intervention in Teenagers
HL: health literacy
IMB: Information-Motivation-Behavioral Skills
PA: physical activity
RCT: randomized controlled trial

## Data Availability

Data sharing is not applicable to this paper, as no data sets will be generated or analyzed during the study.
