# Supplementary material for: A Web-Based Intervention to Improve Health Literacy and Obesogenic Behaviors Among Adolescents: Protocol of a Randomized Pilot Feasibility Study for a Parallel Randomized Controlled Trial
Source: JMIR Res Protoc. 2022 Aug 16;11(8):e40191. doi: 10.2196/40191 (PMC9428776; doi:10.2196/40191)
Supplement: Multimedia Appendix 3 [file resprot_v11i8e40191_app3.pdf]

SUMMARY STATEMENT
(Privileged Communication)

PROGRAM CONTACT:
Dr. LAWRENCE AGODOA
(301) 594-9650
agodoal@extra.niddk.nih.gov

Release Date: 03/30/2019
Revised Date:

Application Number: 1 R21 DK117345-01A1

Principal Investigator

FLEARY, SASHA ALTHEA

Applicant Organization: TUFTS UNIVERSITY MEDFORD

Review Group: ZRG1 DKUS-H (54)
Center for Scientific Review Special Emphasis Panel
PAR-18-102: Small Grants for New Investigators to Promote Diversity in Health-Related Research (R21 Clinical Trial Optional)

Meeting Date: 03/26/2019
Council: MAY 2019
Requested Start: 07/01/2019
RFA/PA: PAR18-102
PCC: MLA SGPD

Project Title: Health Literacy as a Vehicle to Reduce Obesogenic Behaviors Among Adolescents: A Pilot Intervention
SRG Action: Impact Score:24
Next Steps: Visit https://grants.nih.gov/grants/next_steps.htm
Human Subjects: 30-Human subjects involved - Certified, no SRG concerns
Animal Subjects: 10-No live vertebrate animals involved for competing appl.
Gender: 1A-Both genders, scientifically acceptable
Minority: 1A-Minorities and non-minorities, scientifically acceptable
Age: 2A-Only Children, scientifically acceptable

Project Year	Direct Costs Requested	Estimated Total Cost
1	125,000	197,017
2	125,000	197,017
3	125,000	197,017
TOTAL	375,000	591,050

1R21DK117345-01A1 FLEARY, SASHA

RESUME AND SUMMARY OF DISCUSSION: This revised R21 application proposes to examine the effectiveness of adding a health literacy module to an obesity prevention intervention that addresses adolescents' obesogenic behaviors. The reviewers agreed that strengths of the application include the high significance, sound scientific premise supported by the literature and the preliminary studies, strong PI and excellent investigative team, well-described approach, and excellent environment. The recruitment plan for the clinical trial is very detailed and sophisticated, and the activities designed to address the four components of health literacy are comprehensive and interactive. Aim 1 was considered very strong. The PI has been very responsive to the prior review, and the novelty and the scientific rigor of the revised application are significantly improved. There were a few concerns related to the approach and the ability to see differences in the outcomes. These concerns are minor. Overall, the reviewers agreed that this is a very well written application. Enthusiasm by the reviewers for this application was high.

DESCRIPTION (provided by applicant): The prevalence rates of adolescents' obesogenic behaviors are alarming, and reduced life expectancy is the future of America's youth if behavioral changes are not implemented to improve health and reduce the obesity burden. Approximately 69% and 73% of adolescents do not consume daily recommended fruits and vegetables respectively, while 53% engage in insufficient physical activity. These behaviors magnify risks for obesity-related disease burden in adolescence and adulthood including type 2 diabetes, rates of which have increased markedly among adolescents and young adults. Reversing obesity trends requires emphasis on prevention with novel and sustainable intervention strategies to address adolescents' obesogenic behaviors. Predictive theoretical models suggest that health knowledge works in conjunction with motivation and behavioral skills to influence adolescents' obesogenic behavior. However, most existing adolescent interventions target these variables in isolation. Further, health literacy (HL), a precursor to health knowledge, is necessary for translating health knowledge into behavior and is negatively related to adolescents' obesity status. HL is the ability to access, understand, and utilize health information to make informed health decisions. It includes four categories: functional (reading, writing, and numeracy skills), interactive (use health knowledge to communicate and interact with others and environment), critical (advocate for self/others through action), and media (critically analyze health-related media). Yet, HL has not been included in interventions targeting adolescents. We hypothesize that the inclusion of HL into evidence-based interventions will increase intervention effectiveness and behavioral outcomes. Our long-term goal is to reduce the incidence of obesity and chronic disease risk in adolescents through interventions that address individual and contextual factors related to long-term health decision-making and behavior change. The goal of the proposed study is to pilot test the effectiveness of adding a HL module to an obesity prevention intervention that addresses adolescents' obesogenic behaviors. For Aim 1, we will modify successful components from established obesity interventions into an interactive digital platform with and without HL modules. We will test the intervention for accessibility and usability among adolescents 14-16- years-old and modify and retest the intervention iteratively until it is acceptable to adolescents. For Aim 2, the intervention will be piloted among 76 adolescents in a two-arm randomized-controlled-trial to assess preliminary effectiveness and feasibility. The major innovations of the study are the inclusion of HL, a potentially critical factor in adolescents' obesogenic behaviors, and the use of an interactive digital platform for the intervention. We anticipate that the results of this pilot study will lay the groundwork for a future large-scale clinical trial to test the effectiveness of the inclusion of HL on interventions for diverse groups of adolescents.

PUBLIC HEALTH RELEVANCE: The prevalence of adolescent behaviors that can lead to obesity are alarming, and reduced life expectancy is the future of America's youth if behavioral changes are not implemented to improve health and reduce the obesity burden. Researchers have argued that health literacy is a precursor to health knowledge and is necessary for translating knowledge about healthy choices into behavior, with low health literacy being associated with reduced preventive health behaviors in adults. Given the lack of health literacy-specific interventions addressing adolescents'

obesogenic behaviors, the purpose of this study is to examine the preliminary effectiveness of adding a health literacy module to an obesity prevention intervention that addresses adolescents' obesogenic behaviors.

CRITIQUE 1

Significance: 2
Investigator(s): 1
Innovation: 2
Approach: 3
Environment: 1

Overall Impact: This revised application is much improved over the initial proposal. The application retains a focus on health literacy as the key construct of interest, but has completely revise how health literacy is conceptualized and how it will be integrated into a potentially novel intervention. The work will include two phases. In the first phase, an online interactive health behavior change intervention will be developed and refined using cognitive interviews. The intervention is now based on the relational developmental systems framework (RDS) and the Information-Motivation-Behavioral Skills Model (IMBM). She has integrated health literacy into the framework and the result is a much more nuanced understanding of the role of health literacy in behavior change. The core of the intervention draws upon previous work with adolescent behavior change. The novel part of the application is the development of a module to improve health literacy skills. The second phase is a randomized clinical trial (powered to gain information needed to plan a larger clinical) that will compare the health behavior intervention with and without the added health literacy module. The measurement model is now fully specified and will included validated measures of physical activity and dietary behaviors. There is an excellent and detailed recruitment plan and a full clinical trial protocol. This is one of the most improved revised applications I have had the chance to review. The applicant has used the feedback and her local resources to submit a proposal that is novel and potentially significant for public health.

1. Significance:

Strengths

- Poor dietary choices and lack of physical activity put adolescents at risk for weight gain and early development of chronic disease such as diabetes and hypertension. This application seeks to develop a web-based intervention to promote healthy behavior in adolescents and hopes to enhance its effectiveness by building an intervention to improve health literacy. An eHealth application to increase healthy behaviors in adolescents could have significant implications for public health.
- The revised application is now based on a much more sophisticated psychosocial theory and has a good chance of leading to an intervention that will promote healthier behaviors.

Weaknesses

- I still have doubts that health literacy is something easily changed and that it will amplify the effects of a behavioral intervention. However, the study as now proposed will provide important data to answer this question.

2. Investigator(s):

Strengths

- Dr. Fleary is a clinical psychologist who specializes in health behavior and health disparities in adolescents. She has made progress in her development as an academic. She is currently supported by a K-12 award and has continued to publish.
- She is an excellent candidate for this funding mechanism.

- She has clarified the role of her co-investigators and has further documented her ongoing interaction with both.
- She has added a consultant with expertise in health literacy.
- She has a formal arrangement with the Tufts Biostatistics core to receive ongoing consultation on this project, overcoming a criticism of the previous application.

Weaknesses

- None noted.

3. Innovation:

Strengths

- The revised application is far more innovative than the previous version. She will engage with the 3C institute to develop a web-based behavioral intervention that incorporate knowledge, motivation, and behavioral components from the Information-Motivation-Behavioral Skills Model (IMBM). She has also expanded the theoretical framework to better recognize social determinants and other contextual variables. Health Literacy is now viewed as a construct that will enhance the effectiveness of these other evidence-based intervention strategies.
- What is novel is the bringing together of evidence-based behavioral change strategies with an attempt to enhance health literacy.

Weaknesses

- Web-based interventions for adolescents are not novel at this point.

4. Approach:

Strengths

- The two-stage process involving formative development of the intervention followed by a pilot randomized clinical trial is a strength. She will include multiple rounds of cognitive interviews with adolescents to refine the program and has a clear set of standards for judging acceptability.
- She is much more specific about her outcome measures and includes a good plan for capturing eating and physical activity behaviors along with other important constructs.
- She has expanded her network of local organizations and should have a more than adequate pool of adolescents who can be enrolled in the formative work and the clinical trial.
- The design is vastly improved. There will be two groups, evidence-based behavior change and evidence-based behavior change plus health literacy training.
- By using an online web-based platform for intervention, she will be able to collect and analyze high quality process evaluation data.
- The recruitment plan is very detailed and sophisticated using an adaptive framework to monitor and adjust recruitment to meet enrollment goals for sex and ethnicity.

Weaknesses

- If the control group has the health literacy module turned off then the amount of content between the two conditions becomes unbalanced. There should be a replacement module that imparts some knowledge unrelated to health literacy so both interventions have 4 modules with a similar amount of exercise, videos, and other content.
- The intervention modules do not use any self-monitoring with goal setting which is a common component of evidence-based behavior change programs. It seems to rely a great deal on imparting information and not so much on encouraging youth to make changes in daily behaviors.

- In this age group, the weight outcome should be z-BMI or BMI-percentile adjusted for age and sex.

5. Environment:

Strengths

- The environment is very strong. Tufts has many resources to support this work.
- Dr. Fleary has formed excellent relationships with community organizations to support recruitment and enrollment.

Weaknesses

- None noted.

Study Timeline:

Strengths

- There is a complete and detailed clinical trial protocol.
- The timeline is detailed and appears to be realistic.

Weaknesses

- None noted.

Protections for Human Subjects: Acceptable Risks and/or Adequate Protections

- The research is no greater than minimal risk. The risks and benefits are clearly identified and there is a good plan for privacy protection.

Data and Safety Monitoring Plan (Applicable for Clinical Trials Only):

- I think the study needs an internal data safety and monitoring board and not just rely on annual reports to the IRB for the data safety plan. There should be an independent clinician with expertise in adolescent health who can be consulted on safety issues such as adverse events.

Inclusion of Women, Minorities and Children:

- Sex/Gender: Distribution justified scientifically
- Inclusion/Exclusion of Children under 18: Including ages <18; justified scientifically
- The intervention focuses on 14-16 year olds and this is an important age to target for the development of healthy behaviors.

Vertebrate Animals: Not Applicable (No Vertebrate Animals).

Biohazards: Not Applicable (No Biohazards).

Revision:

- This is a vastly improved resubmission.

Resource Sharing Plans: Acceptable

Budget and Period of Support: Recommend as Requested

CRITIQUE 2

Significance: 2
Investigator(s): 2
Innovation: 2
Approach: 3
Environment: 1

Overall Impact: The current proposal addresses adolescent obesity through a technology platform. There is a scientific premise for the study based on previous technology intervention effectiveness. The study team is strong as is the environment. The main innovation is the focus on health literacy. There are minor concerns related to the approach, related to the ability to see differences in the outcomes, and there seems to be a missed opportunity to assess the impact of the different components of health literacy. This is a resubmission that has been very responsive to the initial review and results in a stronger application. Overall, this project has the potential for moderate to high impact.

1. Significance:

Strengths

- The proposal addresses adolescent obesity which is a significant health problem.
- There is a scientific premise for the study in that the intervention is based on successful obesity interventions targeting adolescents, and their theoretical model has also been used to produce significant results.
- The study has the ability to inform on the use of HL as a key factor that may improve treatment outcomes.
- The use of technology as an intervention medium in adolescents is reasonable given their technology use.

Weaknesses

- None noted.

2. Investigator(s):

Strengths

- The PI is qualified to lead the project based on her research in HL and diverse populations. The PI also shows a commitment to research through publications and a progression from institution to federal funding.
- Co-I Nigg provides expertise in behavioral interventions in children.
- Co-I Freund adds expertise in health disparities.
- There is an existing relationship with the technology development partner (3C).
- The team shows a history of working together via publications and grants.

Weaknesses

- None noted.

3. Innovation:

Strengths

- The proposal incorporates HL, which is an emerging area of scientific inquiry.
- The theoretical models are being used to incorporate several levels of the Socioecological Model (though not explicitly referenced).

Weaknesses

- eHealth/mHealth obesity interventions for adolescents are commonplace, though this is not a major limitation.
- The theoretical models seem to repackage existing concepts (e.g. motivation, self-efficacy, autonomy).

4. Approach:

Strengths

- The investigators have conducted preliminary studies in HL that demonstrate HL is associated with obesogenic behaviors.
- There is collaboration with organizations who serve the participants sought for the study.
- The study utilizes a platform that currently hosts other adolescent-focused NIH projects, which shows that the company is familiar with research and behavior change interventions.
- Utilizing data analytics to inform future research design is a strength.
- The activities designed to address the four components of health literacy are comprehensive and interactive.
- There seems to be substantial work that has already been conducted on the intervention based on the LOS from 3C.
- The study uses adequate measures and a design that will allow them to answer the main question so it is generally scientifically rigorous, save for the sample size.

Weaknesses

- It is not clear how the researchers will determine which components from which intervention (Go Girls, DoiT, New Moves) need to be incorporated into their intervention. It is not clear what the successful components of these interventions are and how it was determined. (minor).
- The determination of "acceptable" is vague. For example, on criteria for acceptability is that adolescents must rate it as helpful and likeable. How are these terms defined? What measure will be used to assess these concepts? Are there scores that correspond to "helpful" and "likeable"? Are there means? Median? Mode? Similar questions can be asked of the other "acceptable" criteria. (minor).
- It is not clear why the age range for the adolescents is restricted to 14 – 16 year olds. (minor).
- It is difficult to believe that an n = 76 is powered to determine between group differences in behavioral outcomes for two groups where change is expected in the same direction for both groups. This does not seem like an achievable aim, especially given concerns related to dose strength. (minor).
- This study is centered on the effect of adding HL to an intervention, and given this, HL should be the primary outcome with behavior change as a secondary outcome. Also, it seems as if there should be analyses designed to determine if HL (separately or as a composite) influence the behavior changes. (minor).

5. Environment:

Strengths

- Tufts is an excellent environment for the study. The environment is enhanced via existing relationships with the community partners.

Weaknesses

- None noted.

Study Timeline:

Strengths

- Appropriate.

Weaknesses

- None noted.

Protections for Human Subjects: Acceptable Risks and/or Adequate Protections

Data and Safety Monitoring Plan (Applicable for Clinical Trials Only): Acceptable

Inclusion of Women, Minorities and Children:

- Sex/Gender: Distribution justified scientifically
- Race/Ethnicity: Distribution justified scientifically
- For NIH-Defined Phase III trials, Plans for valid design and analysis: Not applicable
- Inclusion/Exclusion of Children under 18: Including ages <18; justified scientifically

Vertebrate Animals: Not Applicable (No Vertebrate Animals).

Biohazards: Not Applicable (No Biohazards).

Revision:

- The applicant was responsive to reviewers. The theoretical framework was clarified, the design was altered to include a control group, HL was defined, participant pool was widened, etc.

CRITIQUE 3

Significance: 3
Investigator(s): 2
Innovation: 4
Approach: 3
Environment: 2

Overall Impact: The investigators for this study are trying to isolate and better understand the role of health literacy in preventing obesity among adolescents. For Aim 1, they will develop and refine an interactive digital platform that will be capable of giving an obesity prevention intervention to adolescents with and without a health literacy component. In Aim 2, they plan to assess in a pilot RCT the "preliminary effectiveness and feasibility" of the intervention given with and without the health literacy component. They provide reasonably strong evidence that they can recruit and engage an adequate sample for Aims 1 and 2. This is a resubmission. Overall, I think the investigators have been very responsive to the prior reviewers' comments and now submit a well written application and Clinical Trials Protocol. Though I am uncertain if health literacy plays as prominent a role in obesity prevention as the investigators propose, I think this topic is worthy of further investigation. I am enthusiastic that the proposed approach will provide valid data. If the findings are positive, then a definitive RCT would be a reasonable next step.

1. Significance:

Strengths

- Reducing the incidence of obesity and other chronic diseases by improving relevant health behaviors among adolescents is of high significance.
- The role of health literacy as a contributing factor to the obesity epidemic among adolescents is worthy of study.

Weaknesses

- Though health literacy may be an important factor related to obesity, it may be overplayed in this proposal relative to all the other factors that impact obesity. That said, I do think it is worthy of further study.

2. Investigator(s):

Strengths

- The PI is well positioned to lead and complete this research effort. I based this on her prior research productivity and very strong letters of support submitted as part of this application.

- Senior Co-I's and consultants have the skill and experience to provide the additional expertise for the successful completion of this project.

Weaknesses

- None noted.

3. Innovation:

Strengths

- The investigators state the 3 major innovations of the study are the mode of delivery, the theoretical underpinnings of the intervention, and inclusion of health literacy.

Weaknesses

- The electronic format for delivery is relatively new, but not novel.
- Addressing health literacy as a component of a behavioral intervention is not new. Perhaps the way they wish to do so among adolescents is relatively novel.

4. Approach:

Strengths

- The approach, especially for Aim 2, is well described in the PHS Human Subjects and Clinical Trials Information sections.
- RCT is a strength.

Weaknesses

- I would like to have seen more attention to the dietary pattern that will be advocated. In the research strategy section, mention is made of low-fat and fruits and vegetables, but little detail is provided. In the measurement component of the PHS Human Subjects and Clinical Trials Information Section, the following is stated. "Within diet, we are interested in health eating (e.g. fruits and vegetables, whole grain, fiber, calcium) and unhealthy eating (sugary and salty foods, sugar-sweetened beverages, saturated fats)." No mention is made of foods with healthful, but high dietary fat content (e.g. nuts and vegetables oils). How will they be incorporated into the dietary pattern advocated by this intervention?

5. Environment:

Strengths

- Adequate documentation is provided in support of a strong research environment at Tufts relevant to this proposal.
- Letters of support from community partners are strong.

Weaknesses

- None noted.

Study Timeline:

Strengths

- The timeline is appropriate.

Weaknesses

- None noted.

Protections for Human Subjects: Acceptable Risks and/or Adequate Protections

Data and Safety Monitoring Plan (Applicable for Clinical Trials Only): Acceptable

Inclusion of Women, Minorities and Children:

- Sex/Gender: Distribution justified scientifically

- Race/Ethnicity: Distribution justified scientifically
- For NIH-Defined Phase III trials, Plans for valid design and analysis: Not applicable
- Inclusion/Exclusion of Children under 18: Including ages <18; justified scientifically

Vertebrate Animals: Not Applicable (No Vertebrate Animals).

Biohazards: Not Applicable (No Biohazards).

Resubmission:

- As noted under "overall impact," this submission is responsive to previous reviewers' concerns.

Resource Sharing Plans: Acceptable.

Authentication of Key Biological and/or Chemical Resources: Acceptable.

Budget and Period of Support: Recommend as Requested

THE FOLLOWING SECTIONS WERE PREPARED BY THE SCIENTIFIC REVIEW OFFICER TO SUMMARIZE THE OUTCOME OF DISCUSSIONS OF THE REVIEW COMMITTEE, OR REVIEWERS' WRITTEN CRITIQUES, ON THE FOLLOWING ISSUES:

PROTECTION OF HUMAN SUBJECTS: ACCEPTABLE

INCLUSION OF WOMEN PLAN: ACCEPTABLE
INCLUSION OF MINORITIES PLAN: ACCEPTABLE
INCLUSION OF CHILDREN PLAN: ACCEPTABLE

COMMITTEE BUDGET RECOMMENDATIONS: The budget was recommended as requested.

Footnotes for 1 R21 DK117345-01A1; PI Name: Fleary, Sasha Althea

NIH has modified its policy regarding the receipt of resubmissions (amended applications). See Guide Notice NOT-OD-14-074 at http://grants.nih.gov/grants/guide/notice-files/NOT-OD-14-074.html. The impact/priority score is calculated after discussion of an application by averaging the overall scores (1-9) given by all voting reviewers on the committee and multiplying by 10. The criterion scores are submitted prior to the meeting by the individual reviewers assigned to an application, and are not discussed specifically at the review meeting or calculated into the overall impact score. Some applications also receive a percentile ranking. For details on the review process, see http://grants.nih.gov/grants/peer_review_process.htm#scoring.

MEETING ROSTER

The roster for this review meeting is displayed as an aggregated roster that includes reviewers from multiple CSR Special Emphasis Panels of the DKUS aggregate roster for the 2019/05 council round.
This roster for CSR is available at:
http://public.era.nih.gov/pubroster/Reports?DOCTYPE=SEP&DESFORMAT=PDF&AGENDA_SEQ_NUM_P=363929
